# Stigma towards health care providers taking care of COVID-19 patients: A multi-country study

**DOI:** 10.1016/j.heliyon.2022.e09300

**Published:** 2022-04-18

**Authors:** Abdulqadir J. Nashwan, Glenn Ford D. Valdez, Sadeq AL-Fayyadh, Hani Al-Najjar, Hossam Elamir, Muna Barakat, Joseph U. Almazan, Ibtesam O. Jahlan, Hawa Alabdulaziz, Nabil E. Omar, Fade Alawneh, I. Ketut Andika Priastana, Aiman Alhanafi, Bilal Abu-Hussein, Malik Al-Shammari, Marwa M. Shaban, Mostafa Shaban, Hayder AL-Hadrawi, Mohammed B. Al-Jubouri, Sabah A. Jaafar, Shaymaa M. Hussein, Ayat J. Nashwan, Mohammed A. Alharahsheh, Nisha Kader, Majid Alabdulla, Ananth Nazarene, Mohamed A. Yassin, Ralph C. Villar

**Affiliations:** a Department of Nursing Education & Practice Development, Hazm Mebaireek General Hospital (HMGH), Hamad Medical Corporation (HMC), Doha, Qatar; b Faculty of Nursing, University of Calgary in Qatar (UCQ), Doha, Qatar; c Faculty of Nursing, Oman College of Health Sciences, Dhofar Branch, Oman; d Maternal and Child Health Nursing Care Department, College of Nursing, King Saud University, Saudi Arabia; e Maternity and Children Department, Faculty of Nursing, King Abdulaziz University, Saudi Arabia; f Pharmacy Department, National Center for Cancer Care and Research, Hamad Medical Corporation (HMC), Doha, Qatar; g Department of Surgery, King Hussein Cancer Center, Amman, Jordan; h Universitas Triatma Mulya, Indonesia; i Islamic Hospital, Amman, Jordan; j Al Essra Hospital, Amman, Jordan; k Quality and Accreditation Directorate, Ministry of Health, Kuwait; l Ibn Sina Hospital, Ministry of Health, Kuwait; m Community Health Nursing Department, Faculty of Nursing, Cairo University, Cairo, Egypt; n Geriatric Nursing Department, Faculty of Nursing, Cairo University, Cairo, Egypt; o Adult Nursing Department, College of Nursing, The University of Baghdad, Baghdad, Iraq; p College of Nursing, The University of Kufa, Najaf, Iraq; q Adult Nursing Department, College of Nursing, University of Al-Muthanna, Iraq; r Department of Sociology and Social Work, Yarmouk University, Irbid, Jordan; s Mental Health Services (MHS), Hamad Medical Corporation (HMC), Doha, Qatar; t College of Medicine, Qatar University, Doha, Qatar; u Department of Clinical Pharmacy and Therapeutics, Faculty of Pharmacy, Applied Science Private University, Amman, Jordan; v Department of Nursing, Mental Health Services (MHS), Hamad Medical Corporation (HMC), Doha, Qatar; w Department of Medicine, School of Medicine, Nazarbayev University, Nursultan, Kazakhstan; x Department of Medical Oncology, Hamad Medical Corporation (HMC), Doha, Qatar

**Keywords:** Stigma, COVID-19, S19-HCPs, Mental health, Healthcare providers

## Abstract

**Background:**

Health care providers (HCPs) have always been a common target of stigmatization during widespread infections and COVID-19 is not an exception.

**Aim:**

This study aims to investigate the prevalence of stigmatization during the COVID-19 pandemic among HCPs in seven different countries using the Stigma COVID-19 Healthcare Providers tool (S19-HCPs).

**Design:**

Cross-sectional.

**Methods:**

The S19-HCPs is a self-administered online survey (16-item) developed and validated by the research team. The participants were invited to complete an online survey. Data collection started from June–July 2020 using a convenience sample of HCPs from Iraq, Jordan, Egypt, Saudi Arabia, Indonesia, Philippines, and Kuwait.

**Results:**

A total number of 1726 participants were included in the final analysis. The majority of the study participants were Jordanians (22%), followed by Kuwaitis (19%), Filipinos (18%) and the lowest participants were Indonesians (6%). Other nationalities were Iraqis, Saudis, and Egyptians with 15%, 11% and 9% respectively. Among the respondents, 57% have worked either in a COVID-19 designated facility or in a quarantine center and 78% claimed that they had received training for COVID-19. Statistical significance between COVID-19 stigma and demographic variables were found in all aspect of the S19-HCPs.

**Conclusion:**

The findings of this study demonstrated high levels of stigmatization against HCPs in all the included seven countries. On the other hand, they are still perceived positively by their communities and in their utmost, highly motivated to care for COVID-19 patients. Educational and awareness programs could have a crucial role in the solution of stigmatization problems over the world.

## 1 Introduction

In December 2019, the severe acute respiratory syndrome coronavirus-2 (SARS-CoV-2), the virus causing COVID-19 (coronavirus disease 2019), was detected in Wuhan, Hubei Province China, [1]. Since then, according to the World Health Organisation (WHO) statistics, it has spread to over 200 countries with more than 170 million confirmed cases and more than 3.7 million confirmed deaths (as of June 6, 2021). That resulted in panic and fear as government leaders struggled to contain the outbreak through numerous quarantine schemes and lockdowns. Physicians, nurses, and other healthcare providers took the frontlines as part of a massive effort to save as many lives as possible. Healthcare providers have been publicly praised as the heroes of this pandemic. Government leaders and the public have taken over social media to extend their gratitude to health care providers. However, despite the recognition received by health care providers, there have been numerous reports of stigmatization among health care providers worldwide [2, 3, 4].

Health care providers have always been a common target of stigmatization during widespread infections [2]. Stigma is prevalent and well researched concerning mental illness, non-communicable (e.gh, cancers), and communicable diseases like human immunodeficiency virus (HIV), Ebola virus disease (EVD), Tuberculosis (TB) and Severe Acute Respiratory Syndrome (SARS) [5]. People are usually fearful of the unknown, which is the case of COVID-19, a new disease for which there are still many unknowns; social stigma against health care providers who are taking care of COVID-19 patients is highly expected [6]. Historically, the term “stigma” was used to refer to bodily signs designed to expose something unusual and bad about the moral status of the signifier [7]. In present times, stigma is now more related to negative discriminatory thoughts, feelings, and behaviors based on marginalized and racialized identities to those posing certain features that are perceived as unpleasant or threatening [4, 8].

Stigma has many drivers leading to stigmatization, including the fear of infection, blame, stereotypes, social judgment, lack of awareness, fear of social ramifications, and prejudice [9, 10] which mainly fueled by the pandemic of misinformation and linked to certain racial and ethnic groups e.g., ‘Wuhan’ or ‘Chinese’ virus. On the other hand, inadequate and inconclusive research on COVID-19 transmission and preventive measures as well as the chaos of sharing COVID-19 news and updates, raises apprehension among the public, leading to mistrust in healthcare services [9, 11].

Studies about the stigma against health care providers who are taking care of patients with COVID-19 are minimal. According to the WHO [12], the frontline healthcare providers are challenged by stigma, social isolation, and discrimination in the workplace environment and their social surroundings during the pandemic time. Victimization of the frontline health care providers taking care of COVID-19 patients by stigma may negatively impact their focus and decrease the efficiency of making sound decisions [13]. The impact of such circumstances is not just limited to the psychological well-being of health care providers; it can also affects their professional competencies to provide quality care to the population during the pandemic time [13, 14].

The purpose of this study is to investigate the prevalence of stigmatization during the COVID-19 pandemic among healthcare providers in seven different countries using the Stigma COVID-19 Healthcare Providers (S19-HCPs). As of the researcher’s knowledge, this is the first research study investigating stigma among health care providers taking care of COVID-19 patients in multiple countries. The primary objective of this study is to examine the stigma against healthcare providers taking care of COVID-19 patients. In addition, this study will serve as a baseline for future stigma interventions and related education.

## 2 Methods

A cross-sectional design, including a self-administered online survey was used to achieve the study objectives. The S19-HCPs was developed by Nashwan et al (including experts from the mental health services) based on the available literature. The S19-HCPs scale consists of 27 items that are rated and scored differently. Eight items are rated on a three-point Likert scale, and 19 items are rated on a four-point Likert scale. The tool is measuring 6 stigma-related factors including: 1) Fear of getting infected with COVID 19, 2) Following precaution from getting infected with COVID 19, 3) Readiness to care COVID 19 patients, 4) Perception towards the caregivers of the patient with COVID 19, 5) Satisfaction on COVID center’s provisions for safety, and 6) Attitude towards the patient with COVID 19.

The psychometric measures were both tested in English and Arabic. The S19-HCPs’ English and Arabic versions' internal consistency were satisfactory (Cronbach α = 0.79 and 0.74, respectively). Two-week test-retest correlations were all statistically significant for both versions of the tool (ICC = 0.91, *p*0.01; ICC = 0.89, *p*0.01) [15].

The survey link was shared via the appropriate corporate mail group in each participating facility. Data collection started from June 2020 to July 2020 using convenience sampling of health care providers from Iraq, Jordan, Egypt, Kingdom of Saudi Arabia (KSA), Indonesia, Philippines, and Kuwait. In addition, participants were invited to complete an anonymous, voluntarily online survey (QSurvey™).

### 2.1 Ethical approvals

The Ethics Committee approved all study activities of the following Centers:

⁃ Iraq
  - University of Baghdad (UoB) in Iraq (Ref20-09-2020).
⁃ Jordan
  - Jordan University of Science and Technology (JUST) (Ref15-10-2020).
  - King Hussein Cancer Center (KHCC) (04-NOV-2020).
  - Islamic Hospital (6-2020-2968).
  - Israa Hospital (02-01-2021).
⁃ Saudi Arabia
  - Faculty of Nursing, King Abdulaziz University (KAU) (Ref No 2F. 38)
⁃ Kuwait
  - Ministry of Health (1604/2020)
⁃ For the Philippines, Egypt (no IRB approvals were required). For Indonesia, the ethical approval number was obtained from Universitas Triatma Mulya (2020-10022)].

The research information sheet was enclosed with the survey explaining the expectations and study procedure.

## 3 Results

### 3.1 Demographics

A total number of 1726 participants were included in the final analysis. Descriptive statistics were used to describe participants’ characteristics. The majority of the study participants were Jordanians (22%), followed by Kuwaitis (19%), Filipinos (18%) and the lowest number of participants were Indonesians (6%). The prominent study participants are in the age group of 31–40 years (40%) and more than half are females (55%). Most of the study samples were nurses (54%), next to them were physicians (24%), followed by pharmacist (15%) and allied health professionals (8%) (Table 1).

Majority of the participants have less than 6 years of experience in the healthcare field (44%), while health professionals with more than 21 years of experience comprise about 11% of the study participants. In addition, more than half of the study HCPs (57%) have worked either in a COVID-19 designated facility or in a quarantine center. Finally, most of the study samples (78%) have been trained on COVID-19-related stigma and discrimination, Infection control and universal precautions and Patients’ informed consent, privacy, and confidentiality.

### 3.2 Association between occupation of the participants and COVID 19 stigma

The test analysis in Table 3 shows a statistically significance between the health care providers occupation and the study variables: fear of getting infected with COVID-19 (chi square = 19.951, p < 0.0001), perceptions towards caregivers (chi square = 14.033, p = 0.003), and attitude towards COVID-19 patients (chi square = 33.805, p = 0.000). The physicians among other healthcare providers possessed high levels of fear of getting infected with COVID-19 infection with the mean of 956.84 while the other group of healthcare providers allied health and pharmacist have comparatively merely less and same level of fear score (mean of 820.59 and 821.94 respectively). The researchers can conclude that allied health healthcare providers are perceived better while caring the patients with COVID-19 with a high mean of 909.18 compared to other healthcare providers like nurses with the mean score of 891.77 and physicians who were perceived comparatively low with a mean of 787.16. In addition, the nurses are noted to have the highest attitude mean score of 919.21.

### 3.3 Association between gender of the participants and COVID 19 stigma

The association between healthcare provider’s gender and satisfaction on the safety provisions in the COVID-19 centers is statistically significant (see Table 3) (chi square = 15.284, and p = 0.000). The male healthcare providers are with the high mean of 913.14, followed by female healthcare providers (824.3) and healthcare providers who don’t want to reveal their gender (776.94). The researchers can conclude that male healthcare providers are more satisfied on the provision of COVID-19 centers for safety compared to other genders.

The researchers also identified a statistical significance between gender and their attitude towards the patients with COVID-19 infection with the chi square of 20.124 and p = 0.000 (see Table 3). The healthcare providers who don’t want to reveal their gender noted to have high attitude mean score of 1108.17 while the female healthcare providers exhibit low attitude score with the mean score of 824.43, meanwhile the male healthcare providers scored the medium score of (918.21) attitude towards the patients with COVID-19 infection (see Table 1).

**Table 1:** Participants’ characteristics

Variable	N	%
**Occupation**		
Physician	405	23.5
Nurse	932	54.0
Pharmacist	250	14.5
Allied health	139	8.1
**Gender**		
Male	765	44.3
Female	952	55.2
I don’t want to disclose	9	.5
**Age**		
20–30yrs	627	36.3
31–40yrs	698	40.4
41–50yrs	282	16.3
51+	119	6.9
**Years of experience in Healthcare**		
1–5yrs	766	44.4
6–10yrs	300	17.4
11–20yrs	467	27.1
21+	193	11.2
**Worked in a COVID19 facility or Quarantine centers**		
Yes	989	57.3
No	737	42.7
**Training**		
Yes	1348	78.1
No	378	21.9
**Nationality**		
Iraq	251	14.5
Jordan	385	22.3
Egypt	151	8.7
KSA	187	10.8
Indonesia	104	6.0
Philippines	320	18.5
Kuwait	328	19.0

### 3.4 Association between age of the participants and COVID-19 stigma

The results in Table 3 depicts that the age of the healthcare providers are statistically significant with their readiness to care for COVID-19 patients (chi square = 15.126, with p = 0.002), perception towards the healthcare providers of COVID-19 (chi square = 33.576, with p = 0.000), satisfaction on the safety provisions in the COVID centers (chi square = 12.896, with p = 0.005), and attitude towards the patients with COVID-19 infection (chi square of 32.090 and p = 0.000). Healthcare providers aged between 20-30 years has the highest mean scores in all the variables compared to healthcare providers who are more than 50 years who obtained the lowest mean scores (see Table 2). The researchers can conclude that the younger healthcare providers tend to have a better outlook when it comes to the perception towards them by the society, safety provisions by COVID centers, and their readiness to care for patients with COVID-19.

**Table 2:** Variables and difference in mean COVID-19 stigma scores.

		Fear of getting infected with COVID 19	Following precaution from getting infected with COVID 19	Readiness to care COVID 19 patients	Perception towards the caregivers of the patient with COVID 19	Satisfaction on COVID center’s provisions for safety	Attitude towards the patient with COVID 19
**Mean Scores**							

Occupation	physician	956.84	877.86	823.95	787.16	826.57	753.77
	nurse	840.49	879.98	867.92	891.77	888.63	919.21
	pharmacist	821.94	804.83	903.52	856.39	826.51	824.88
	allied health	820.59	816.69	877.09	909.18	869.15	879.17
Gender	male	867.51	866.00	886.42	873.46	913.14	918.21
	female	860.48	863.38	846.03	855.56	824.43	817.23
	I don’t want to disclose	842.56	664.00	763.17	856.67	776.94	1108.17
Age	20–30yrs	840.42	885.35	923.20	949.93	913.59	920.11
	31–40yrs	856.49	831.92	838.55	833.58	846.29	873.56
	41–50yrs	923.11	907.86	819.74	789.54	827.93	722.34
	51+	884.97	828.52	798.98	758.85	784.80	840.71
Years of experience in Healthcare	1–5yrs	1006.29	1016.54	1026.71	1085.28	920.21	847.65
	6–10yrs	728.09	741.30	775.69	752.26	884.70	932.10
	11–20yrs	763.43	751.68	726.76	677.41	808.81	853.36
	21 + yrs	749.39	716.59	683.11	606.47	737.81	844.31
Worked in a COVID-19 designated facility or Quarantine centers	Yes	944.22	953.75	945.63	1007.92	879.73	880.03
	No	755.18	742.40	753.29	669.70	841.72	841.32
Training	Yes	894.03	899.26	884.51	893.00	844.24	838.79
	No	754.63	735.99	788.56	758.30	932.18	951.62
Nationality	Iraq	728.82	793.83	879.89	925.80	1127.52	1184.58
	Jordan	686.89	736.04	834.19	762.19	738.62	903.78
	Egypt	668.09	719.17	910.34	726.69	993.32	924.80
	KSA	741.93	744.04	576.15	540.06	652.44	765.75
	Indonesia	666.57	813.94	695.30	681.43	664.35	1169.50
	Philippines	1446.11	1379.17	1305.18	1473.82	1000.90	647.60
	Kuwait	827.17	713.61	650.06	644.42	797.70	711.64

### 3.5 Association between years of healthcare experience and COVID 19 stigma

The results in Table 3 illicit that there is statistically significance found between years of experience in healthcare and fear of getting infected with COVID-19 (chi square = 119.929, with p = 0.000), readiness to care COVID-19 patients (chi square = 154.051, with p = 0.000), perception towards them while caring the COVID 19 patients (chi square = 289.513, with p = 0.000), and satisfaction on the safety provisions in COVID-19 centers (chi square = 31.613, p = 0.000). The healthcare providers with 1–5yrs of healthcare experience possessed high levels of fear of COVID-19 with the mean of 1006.29 and the lowest mean score were among the healthcare providers with 6–10yrs experience (728.09). Interestingly, despite the less experienced healthcare providers’ high levels of fear, they showed the highest level of readiness to care of COVID-19 patients (1026.71) compared to well experienced healthcare providers (see Table 2).

**Table 3:** Test Statistics between study variables and demographic variables.

Association between variables of the participants and COVID 19 stigma		Fear of getting infected with COVID 19	Following precaution from getting infected with COVID 19	Readiness to care COVID 19 patients	Perception towards the caregivers of the patient with COVID 19	Satisfaction on COVID center’s provisions for safety	Attitude towards the patient with COVID 19
Occupation	Chi-Square	19.951	8.113	4.401	14.033	6.674	33.805
	df	3	3	3	3	3	3
	Asymp. Sig.	.000	.044	.221	.003	.083	.000
Gender	Chi-Square	.106	1.961	3.196	.562	15.284	20.124
	df	2	2	2	2	2	2
	Asymp. Sig	.949	.375	.202	.755	.000	.000
Age	Chi-Square	6.038	9.164	15.126	33.576	12.896	32.090
	df	3	3	3	3	3	3
	Asymp. Sig	.110	.027	.002	.000	.005	.000
Years of healthcare experience	Chi-Square	119.929	175.194	154.051	289.513	31.613	7.125
	df	3	3	3	3	3	3
	Asymp. Sig.	.000	.000	.000	.000	.000	.068
Working status at COVID facility	Chi-Square	63.929	101.920	63.788	198.917	2.738	2.617
	df	1	1	1	1	1	1
	Asymp. Sig.	.000	.000	.000	.000	.098	.106
Training	Chi-Square	24.303	42.517	11.097	22.057	10.246	15.539
	df	1	1	1	1	1	1
	Asymp. Sig.	.000	.000	.001	.000	.001	.000
Nationality	Chi-Square	251.749	464.367	115.293	181.165	564.414	252.530
	df	6	6	6	6	6	6
	Asymp. Sig.	.000	.000	.000	.000	.000	.000

Furthermore, the healthcare providers with 1–5yrs of experience feel that they are perceived well while caring the patients with COVID-19 with merely high mean score of 1085.28 and are more satisfied with the safety provisions in COVID-19 centers (920.21) compared to healthcare providers with 21 + yrs of experience with the lowest score (see Table 2).

### 3.6 Association between participants working status at a COVID facility and COVID-19 stigma

The test analysis illicit that there is statistically significance found between the working status of the healthcare providers at the COVID facility or quarantine centers and fear of getting infected with COVID 19 (chi square = 63.929, p = 0.000), willingness to follow the precautions (chi square = 101.920, with p = 0.000), readiness to care for COVID-19 patients (chi square = 63.788, with p = 0.000), and perception towards healthcare providers (chi square = 198.917, with p = 0.000). The healthcare providers who worked in a COVID-19 designated facility or quarantine centers possessed high levels of fear of COVID-19 with the mean of 944.22. They also achieved the highest mean scores in following precautions and readiness to care for COVID-19 patients. Healthcare providers who worked in a COVID-19 designated facility or quarantine centers are also perceived well while caring the patients with COVID-19 with high mean score of 1007.92, compared to healthcare providers who did not worked in a COVID-19 designated facility or quarantine centers scored low (606.47) (see Table 2).

### 3.7 Association between healthcare providers training status and COVID-19 stigma

The test analysis in Table 3 illicit that there is a statistically significance found between training status of healthcare providers with fear of getting infected with COVID 19 (chi square = 24.303, with p = 0.000), willingness to follow the precautions against COVID-19 (chi square = 42.517, with p = 0.000), perception towards healthcare providers caring for COVID-19 patients (chi square = 22.057, with p = 0.000), satisfaction on the safety provisions in the COVID centers (chi square = 10.246, and p = 0.001) and attitude towards the patients with COVID-19 infection (chi square of 15.539 and p = 0.000).

The healthcare providers who were trained possessed high levels of fear of COVID-19 infection with the mean of 894.03 compared to the healthcare providers who did not get the training (754.63). The healthcare providers who were also trained are more willing to follow precautions (899.26) and are perceived better by the society (893.00) compared to healthcare providers who were not trained (see Table 2).

Interestingly, the healthcare providers who were not trained are more satisfied with the safety provisions (932.18) and have a better attitude towards patients with COVID-19 (951.62) compared to healthcare providers were trained (see Table 2).

### 3.8 Association between nationality and COVID-19 stigma

Among the demographic variables, only the healthcare providers' nationality showed statistical significance in all study variables. Table 3 reveals that the healthcare providers’ nationality is statistically significant with fear of COVID-19 (chi square = 585.364, with p = 0.000), willingness to follow the precautionary measures (chi square = 572.746, with p = 0.000), readiness to care for COVID-19 patients (chi square = 393.937, with p = 0.000), perception towards them is statistically significant (chi square = 682.291, with p = 0.000), satisfaction on the safety provisions in the COVID centers is statistically significant (chi square = 206.272 and p = 0.000), and attitude towards the patients with COVID 19 infection (chi square of 252.530 and p = 0.000).

The levels of fear were higher among the Filipino healthcare providers with the mean of 1446.11 and the lowest fear of getting infected with COVID-19 was registered among Indonesian healthcare providers with the mean of 666.57. Despite the level of fear, Filipino healthcare providers showed the highest score in their readiness to care for COVID-19 patients. Furthermore, Filipino healthcare providers think that they are perceived well while caring for COVID-19 patients and they also obtained the highest mean score in the willingness to follow precautionary measures (1379.17).

Meanwhile, Iraqi healthcare providers are more satisfied with the safety provisions of COVID-19 centers having the highest mean score of 1127.52 while healthcare providers of Saudi nationality have the lowest mean score of 652.44. The Iraqi healthcare providers noted to have the highest attitude mean score of 1184.58 compared to Filipino healthcare providers who has the lowest attitude mean score of 647.60.

## 4 Discussion

### 4.1 A multi-country perspective

The study objectives were met by investigating the prevalence of stigmatization during the COVID-19 pandemic among 1,726 healthcare workers using the self-developed COVID-19 Healthcare Providers tool (S19-HCPs) in seven different countries. The demographic profiles show that Jordanian, Kuwaitis, and Filipino top the list of the participants that are young adults, females and comprised mainly of healthcare workers in the profession of nursing, medicine, and pharmacy. The majority of which had five years of experience, assigned in a COVID-19 facility, and underwent the training on a COVID-19 stigma, discrimination, infection control, universal precautions, patients' informed consent, privacy, and confidentiality. Our study revealed that there is a high level of fear of being infected among HCPs. On the other hand, the level of readiness to care and perception towards caregivers of patients for COVID-19 are high, while the satisfaction on COVID-19 provision for safety and attitude towards patients were relatively moderate among genders but high among nurses and younger HCPs.

### 4.2 Fear of getting infected with COVID-19

COVID-19 has given rise to fear among the public and is more so to the HCPs handling the patients directly. A global survey among healthcare workers and the public showed a great sense of loss of control of becoming infected and dying, more so infecting their loved ones with COVID-19 [16], although fear can also be attributed to decreased physical and environmental well-being [17, 18]. COVID-19 has posed a serious occupational health risk to HCPs due to their frequent exposure to infected individuals [19, 20]. Fear among HCPs in countries where COVID-19 is on a steady rise can be among the many factors contributing to their worries. In the recent statistics from the WHO, as of to date June 2021, 174,061,995 million people around the globe was infected by the virus, countries such as India, Indonesia, and the Philippines were among the highest in Asia [21]. Based on the study results, the Filipino respondents recorded the highest fear of getting infected. However, fear among healthcare workers was seen as universal among other countries [22, 23], fear can also gravely affect their performance at work and the way they deal with the patient, which can lead further to burnout and anxiety that is detrimental as to how they perform their duties [24, 25]. The case of Filipino healthcare worker is unique considering that vast majority of its healthcare workforce are scattered in the diaspora. Undeterred by the pandemic, many Filipinos continue to emigrate to join the healthcare industry abroad [26]. The sense of fear is magnified by several contributing factors such that of being miles away from their families, working in a foreign environment, lack support, the rising death tolls of frontline healthcare workers at home and overseas and the obligation to follow their employers as immigrants on visas [27]. Curiously, other frontline HCPs caring for COVID-19 patients are described to have less fear about becoming infected than HCPs in other units, like the study conducted about the fears related to SARS that have similar results [28]. The study findings also suggest that age, gender, type of occupation is significant in the level of fears felt by the healthcare workers. This study identified older and female healthcare workers tend to be more fearful of being infected and like those recently published [29]. Physicians possessed the highest level of fear on being infected. Fear stems not only from personal reasons or loss of health integrity but also brought by the rising concerns of how HCP are perceived publicly; there are growing accounts of HCPs being spurned and hounded by a fearful public because of their occupation [30]. A global study among HCPs revealed they significantly experienced more COVID-19-related bullying after identifying the confounding effects of job-related, personal, geographic, and sociocultural variables [31]. However, not all stories about HCPs are negative, several stories are positive, encouraging and talks about their bravery; HCPs honors us all with their commitment, dedication, and professionalism [32].

### 4.3 Following precautions from getting infected with COVID-19

Moreover, fear may emanate from several factors other than being exposed to COVID-19; various mechanisms may prevent infection among healthcare workers deemed necessary to keep the health sector from reaching its limit. Preventive measures are essential in maintaining a steady flow of workers and not adding up to the already piled up patients in the COVID-19 wards. In this study, physicians and nurses are highly compliant with the preventive measures. However, compliance with strictly adhering to preventive measures may vary according many factors such as to the healthcare workers nature of contact with the patients; professions such as pharmacist are the least likely to follow the precautionary measures since they are less exposed to the infected patients, according to a study conducted in Ghana [33]. In addition, many previous studies showed that compliance with standard precautions guidelines is low among health care workers [34, 35].

Most parts not adhering to precautionary measures may stem from the lack of materials such as personal protective equipment (PPE). A South American study on PPE showed that the lack of this equipments had caused the rise of infection among healthcare workers [36]. In the early stages of the pandemic, panic of buying PPE’s has left healthcare workers ill-equipped to take care of the sick, there was a mounting disruption in the supply of the equipment, The World Health Organization (WHO) has stepped up to deliver the much needed protective materials to the countries in need countries in the Middle East, Africa, and Asia Pacific (WHO, Shortage of personal protective equipment endangering health workers worldwide, 2020). The lack of PPE’s and Non -adherence to the set precautionary protocols often leads to HCPs being infected some even succumbs into the COVID-19 infection. The rise of HCPs COVID -19 infection and mortality in the early stages of the pandemic has left most health care system struggling; high rates of morbidity and mortality in elderly healthcare workers may require assigning them to less risky settings such as telemedicine, non- COVID-19 outpatient clinics, or administrative positions [37].

### 4.4 Readiness to care for COVID-19 patients

Furthermore, HCPs continuously hold the line by taking care of the sick and infected. This was evident in the study that although faced with the risk, healthcare workers show a high level of readiness in the care of COVID-19 patients, especially among younger health workers who are currently assigned in COVID-19 facilities. This is consistent with the study of [38, 39, 40], which mentioned that readiness requires adequate information, training, and workplace practice. Pandemic information is crucial to HCPs' readiness, with the abundance of misleading knowledge from unknown sources often leads to prejudice as already advised by scientists and WHO officials [41]. The HCPs must seek valid and important information that is factual and verified; there is an overabundance of malicious, fake information on the internet that could misguide the HCPs. This situation, in turn, demands careful screening and verification of information sources [42]. Appropriate protection can only be maintained if coupled with training and experience on the same premise; the management of COVID-19 patients requires extensive training and practice to lower the risk of HCPs being infected. The study findings revealed that HCP’s who have been trained and exposed to handling COVID-19 patients are confident in being assigned in COVID -19 centers, contrary to those who are not assigned to care for COVID-19 patients directly. Readiness training includes that of proper PPE management by using personal protective equipment appropriate for SARS-CoV-2 consists of protective masks, round caps, gloves, protective clothing, boot covers, and goggles or a face shield, and the proper use of this protection decreases the risk of infection among healthcare professionals and was considerably reduced, though not eliminated [43]. On top of the physical training and preparation, most healthcare institutions have started introducing the concepts related to stigma, discrimination, informed consent, privacy, and confidentiality as a part of the training and orientation programs. These programs and training resulted from the number of reported cases of discrimination among healthcare workers worldwide during the peak of the COVID-19 pandemic. For example, in the Philippines, a health care worker was attacked, evicted from their rental homes, harassed, and ridiculed in their homes and workplaces were a trending topic in the height of the pandemic [44]. Similar incidents were reported in India, the USA, and Australia, where they are even being beaten, threatened and removed from their homes [45].

Additionally, HCPs in a study done in Nepal were treated as untouchables and are being set apart, have lost status because of the stigma associated with COVID-19 [13]. Although there were no reports in most participating countries in this study, it is imperative to know that discrimination and stigmatization can be associated with societal structure, background, and culture. Propagating truthful information may help neutralize COVID-19 stigma in people and eliminate social discrimination the frontline healthcare providers are facing, which will safeguard their mental well-being and help regulate this public health crisis effectively [13].

### 4.5 Perception towards the caregivers of patients with COVID-19

On the perception of caregivers for patients with COVID-19, the HCPs who were Filipino by nationality, young adults ages 20–30, working as nurses and as allied health professionals, trained in the care of COVID-19 patients perceived themselves well received by the caregivers of their patients. Caregivers, primarily family members and relatives, play a crucial role in caring for patients with COVID-19 as support systems. When a COVID-19 patient is admitted, they are usually left alone in the care of the HCPs. In most situations, the family members rely heavily on the HCPs about the care and communication of their condition to them. On many occasions in this pandemic, the HCP’s who are with the patients till their last breaths; it is a situation that has put pressure, fear, and agony among the HCP and their family members. Given these circumstances, HCP has been creative and innovative enough to find ways to involve family members in the care of their COVID-19 patients who are in the ICU or nursing homes by using mobile devices and mobile applications to bridge the communication barriers [46]. Contrary to this, some experiences are considered hurtful among HCPs when family members and caregivers themselves discriminate against them through hurtful words, vulgar comments, or false comments about the care of patients [47].

### 4.6 Satisfaction on COVID centers provisions for safety

Satisfaction on the safety provided by COVID-19 centers reveals that Iraqi & Filipino, Male, Nurses who at least have 1–5 years of experience and was trained in COVID quarantine centers were highly satisfied with the safety of the COVID-19 centers. This satisfaction implies a steady supply of PPE’s and well-managed protocols in infection control fully understood and implemented in the participants' respective COVID-19 treatment facility assignment. On the contrary, in most parts of the world, healthcare workers rely heavily on personal protective equipment to protect themselves while caring for COVID-19 patients [48]. At the onset of the pandemic, the virulence and infectiousness of the disease have affected the demand for PPE’s supply chain has significantly impacted the HCP’s ability to treat their patients and protect themselves [49]. The drastic demand has led to shortages in countries where there is a surge in COVID 19 cases; in six countries, less access to PPE was considerably associated with both heightened risk of reporting COVID-19 illness as well as more sustained and severe disease course in frontline HCPs [50]. While suggestively the judicious use of PPE to reduce the incidence of the COVID-19 infection to a bare minimum in healthcare settings in countries like India [51].

Furthermore, regulatory bodies like the Center for Disease Control and Prevention (CDC) provided strategies to offer a continuum of options when the supply for personal protective equipment is stressed, running low, or absent. The quality of PPE’s is also an issue that needs to be addressed as evidence showed that total body covering and higher specifications of mask and respirators provide better protection for HCP’s coupled with the skills in donning and doffing is seen as important [52]. Moreover, more stringent studies may be required to support the effectiveness of using PPE’s in decreasing the number of HCPs COVID-19 infection rates.

### 4.7 Attitudes towards patients with COVID-19

HCPs exhibited a positive attitude towards patients, which is evidently high among nurses, the younger age group, and those who were trained in COVID-19 facilities. The stigmatization of COVID-19 in the community is also highly evident in the work environment and facilities catering to infected patients. Attitude and perception of HCPs towards the disease condition may somehow affect the quality of care being given to their clients. There is insufficient knowledge and a low positive attitude among HCPs dealing with COVID19 patients [40, 53]. This contrasts with the study of [54, 55] that yield positive attitude towards COVID 19 patients and the need for a better understanding the course of the disease through reliable information sources and that male with A higher level of degree tends to be more positive [56]. Albeit all the information drives against discrimination in countries around the world like India and the Philippines, cases of misinformed individuals have cause uproar among frontline workers to heed their call for better treatment and to end discrimination [13, 47]. Mitigating the effects of discrimination and the call for cohesiveness among HCP’s is an important step in reducing the stigma around this pandemic, its prevention, and containment; that might be able to develop immediate and long-term strategies to build empathy for those inflicted with this disease by amplifying voices, acting responsibly and wording sensitively [32].

### 4.8 Study implications

Now more than ever, the significance of HCP’s was fully recognized. The insurmountable dedication given by the HCP’s made it possible for the healthcare system around the world not to succumb to COVID-19. However, the pandemic is far from over as to date Globally, as of 11:32 am CEST, 17 June 2021, 176,531,710 confirmed cases of COVID-19, including 3,826,181 deaths, reported to WHO [21]. Our study provided a better understanding of the stigma and discrimination faced by HCP’s in different countries. These documented circumstances from the participants' perspective and the accumulated works of literature imply a better and more systematized management of stigmatization and discrimination in the workplace and in the community at large. Its recommended stringent measures can safeguard the HCP’s from unwanted acts towards them as they care for COVID-19 patients. Public awareness about the COVID-19 must be strengthened by providing reliable sources that may help in educating the public about the facts relevant for better understanding the disease. The provision of a safe workplace equipped with the necessary paraphernalia is an utmost priority among HCP’s, for plans will demand healthcare institutions, global and national health agencies to be prepared in handling such a massive health crisis. The HCP’s shaped and sharpened by this pandemic are a testament to the resiliency, dedication of this era’s unsung heroes.

### 4.9 Limitations

The present study has some limitations. 1) the survey was disseminated electronically via numerous social media platforms, which in one way or another, limiting participation for only those who had an active email account and access to the internet, which raises a possibility of selection bias. 2) the survey was conducted over several months during several waves of the pandemic, and the respondents' perceptions may change over time, with a possibility of recall bias. Finally, 3) this study has followed a convenient sampling technique and difficulty to estimate the sample size where the results might not apply to all other countries.

## 5 Conclusions

Based on the preceding study findings, it can be concluded that participants from the seven countries overall, although having perceived high levels of stigmatization, still observe positively by their respective communities and, in their utmost, highly motivated to care for COVID-19 patients. There are still lurking fears of discrimination among HCPs. Therefore, the fear of getting infected with COVID-19 is proportionally significant among respondents when grouped according to nationality, age, work status, and training status. Preventive measures are followed stringently by healthcare providers that are trained in caring for COVID-19 patients. Summarily, participants have a high readiness to care for COVID-19 patients, perceived positively by caregivers and relatives, highly satisfied with the safety measures accorded by their respective facilities, and have a high positive attitude towards COVID-19 patients. These indicators reflect the overall improvement in the protocols relevant to COVID-19 care one year past the emergence of the disease.

## Declarations

### Author contribution statement

Abdulqadir J. Nashwan, Glenn Ford D. Valdez, Sadeq AL-Fayyadh, Hani Al-Najjar, Hossam Elamir, Muna Barakat, Joseph U. Almazan, Ibtesam O. Jahlan, Hawa Alabdulaziz, Nabil E. Omar, Fade Alawneh, I. Ketut Andika Priastana, Aiman Alhanafi, Bilal Abu-Hussein, Malik Al-Shammari, Marwa M. Shaban, Mostafa Shaban, Hayder AL-Hadrawi, Mohammed B. Al-Jubouri, Sabah A. Jaafar, Shaymaa M. Hussein, Ayat J. Nashwan, Mohammed A. Alharahsheh, Nisha Kader, Majid Alabdulla, Ananth Nazarene, Mohamed A. Yassin, Ralph C. Villar: Conceived and designed the experiments; Performed the experiments; Analyzed and interpreted the data; Contributed reagents, materials, analysis tools or data; Wrote the paper.

### Funding statement

This work was supported by Qatar National Library.

### Data availability statement

Data included in article/supplementary material/referenced in article.

### Declaration of interests statement

The authors declare no conflict of interest.

### Additional information

No additional information is available for this paper.
